# The ACE2/Ang-(1-7)/MasR axis alleviates brain injury after cardiopulmonary resuscitation in rabbits by activating PI3K/Akt signaling

**DOI:** 10.1515/tnsci-2022-0334

**Published:** 2024-04-11

**Authors:** Jing Cheng, Hong Yang, Fang Chen, Li Qiu, Fang Chen, Yanhua Du, Xiangping Meng

**Affiliations:** Department of Emergency, Wuhan Fourth Hospital, Wuhan 430030, China; General Practice Ward, Wuhan Fourth Hospital, No. 473 Hanzheng Street, Qiaokou District, Wuhan 430030, Hubei, China

**Keywords:** cardiac arrest, cardiopulmonary resuscitation, brain injury, ACE2/Ang-(1-7)/MasR axis, PI3K/Akt, apoptosis

## Abstract

**Background:**

Death among resuscitated patients is mainly caused by brain injury after cardiac arrest/cardiopulmonary resuscitation (CA/CPR). The angiotensin converting enzyme 2 (ACE2)/angiotensin (Ang)-(1-7)/Mas receptor (MasR) axis has beneficial effects on brain injury. Therefore, we examined the roles of the ACE2/Ang-(1-7)/MasR axis in brain injury after CA/CPR.

**Method:**

We used a total of 76 male New Zealand rabbits, among which 10 rabbits underwent sham operation and 66 rabbits received CA/CPR. Neurological functions were determined by assessing serum levels of neuron-specific enolase and S100 calcium-binding protein B and neurological deficit scores. Brain water content was estimated. Neuronal apoptosis in the hippocampus was assessed by terminal deoxynucleotidyl transferase dUTP nick end labeling assays. The expression levels of various genes were measured by enzyme-linked immunosorbent assay and western blotting.

**Results:**

Ang-(1-7) (MasR activator) alleviated CA/CPR-induced neurological deficits, brain edema, and neuronal damage, and A779 (MasR antagonist) had the opposite functions. The stimulation of ACE2/Ang-(1-7)/MasR inactivated the ACE/Ang II/AT1R axis and activated PI3K/Akt signaling. Inhibiting PI3K/Akt signaling inhibited Ang-(1-7)-mediated protection against brain damage after CA/CPR.

**Conclusion:**

Collectively, the ACE2/Ang-(1-7)/MasR axis alleviates CA/CPR-induced brain injury through attenuating hippocampal neuronal apoptosis by activating PI3K/Akt signaling.

## 1 Introduction

Cardiac arrest (CA) is a predominant life-threatening circumstance. Cardiopulmonary resuscitation (CPR) offers a crucial medical intervention for patients with CA [1,2], and improves the survival of experimental models of CA [3,4]. However, parallel brain injury leads to unsatisfactory recovery. As reported, 80% of survivors enter a comatose state, 40% remain in a persistent vegetative state [5], and 40–50% of patients die within 6 months [6]. Brain injury is the primary causative factor of death among resuscitated patients [7]. Cell apoptosis is involved in brain injury following resuscitation [8,9]. Thus, a better understanding of the underlying mechanisms of CA/CPR and effective pretreatments to protect against brain injury by preventing apoptosis are critical.

The renin–angiotensin–aldosterone (RAS) system is involved in the regulation of physiology and homeostasis in the cardiovascular system, and is responsible for the maintenance of vascular tone by regulating extracellular fluid volume and blood pressure [10]. The RAS is a complex endocrine, paracrine, and autocrine system with many components [11]. It is currently accepted the existence of two different branches of RAS: the classical angiotensin-converting enzyme (ACE)/angiotensin (Ang) II/angiotensin type 1 receptor (AT1R) axis and the counterregulatory arm ACE2/angiotensin (Ang)-(1-7)/mas receptor (MasR) axis. Studies have confirmed that these two axes in the brain tissue can exert neuronal damage and neuroprotection, respectively [12,13]. Ang I is transformed to Ang II by ACE, and AT1R is a major Ang II receptor. The ACE/Ang II/AT1R axis can regulate hemodynamics and maintain ventricular remodeling and myocardial cell apoptosis [14,15]. Activated Ang II signaling and AT1R overproduction contribute to stroke pathophysiology [16,17]. Ang-(1-7) is formed by ACE2 and is a heptapeptide product of Ang 1/II, and its receptor is MasR. The ACE2/Ang-(1-7)/MasR axis can beneficially influence the outcomes of experimental stroke models [18–20]. Additionally, this axis prevents myocardial infarction or ischemic reperfusion by attenuating myocardial cell apoptosis [21], improves right heart function after acute pulmonary embolism [22], and prevents myocardial ischemia/reperfusion damage [23]. Moreover, its activation during electropuncture protects against memory impairments and anxiety-like symptoms [24], attenuates cell death following glucose deprivation, and decreases infarct volume in a cerebral ischemia‒reperfusion model [25]. However, the roles of ACE2/Ang-(1-7)/MasR in brain injury following CA/CPR remain unclear.

Accumulating evidence has suggested that the PI3K family is associated with cell survival, metabolism, and migration [26]. The stimulation of PI3K/Akt pathway can repress apoptosis and neuroinflammation induced by systematic ischemia/reperfusion injury following CA/CPR [27]. Exogenous Ang-(1-7) administration can prevent germ cell apoptosis by activating PI3K/Akt signaling [28]. Additionally, the upregulation of Ang-(1-7)/MasR can decrease the proapoptotic signaling cascade through the induction of Akt phosphorylation [29]. Therefore, this study was designed to explore the roles and related mechanisms of ACE2/Ang-(1-7)/MasR axis in brain injury after CA/CPR. We believe that this study will provide a theoretical basis for future research on brain injury after CA/CPR and facilitate the development of effective therapeutic options, which has important clinical significance for improving the prognosis of patients with CA/CPR.

## 2 Methods

### 2.1 Animals

Seventy-six male New Zealand rabbits (2 kg of weight) were provided by the Charles River Laboratories (Beijing, China). The rabbits were housed at 18–24°C and underwent an overnight fast prior to the experiment.

### 2.2 CA/CPR models

The CA/CPR model was established as previously described [30]. After being fasted overnight and anesthetized with intramuscular injection of 50 mg/kg ketamine and 4 mg/kg xylazine, the rabbits were fixed in a supine position with their extremities immobilized. The rabbits were tracheotomized for endotracheal intubation. Polyethylene-50 catheters were inserted into the left femoral vein and artery for drug administration and blood pressure measurement, respectively. Subcutaneous needle electrodes were used to monitor and record the electrocardiograms. After the mean arterial pressure (MAP) was over 60 mmHg, oxygen-deficient CA was induced by the injection of KCl (5 mL of 0.5 mol/L) and tracheal tube occlusion (CA indicator: MAP <25 mmHg). The dose of KCl was determined as previously described [31].

CPR was induced by chest compressions (300 bpm) and mechanical ventilation (190 breaths/min) after 6 min of CA. Return of spontaneous circulation (ROSC) was defined by a MAP >60 mmHg for 10 min. If resuscitation failed within 2 min, the rabbits were administered epinephrine (20 μg/kg) for 3 min with precordial compressions for several minutes. Resuscitation efforts were discontinued if ROSC failed within 15 min, and the animals were excluded. Mechanical ventilation was withdrawn after ROSC, and all catheters were removed. Sham animals underwent the same procedures except KCl injection, epinephrine administration, and chest compressions. The rabbits were subsequently returned to their home cages for survival follow-up. The rabbits received intramuscular injections of antibiotics (enrofloxacin, 5 mg/kg) and subcutaneous injections of analgesics (buprenorphine, 30 μg/kg) [32]. The rabbits were euthanized using 300 mg of intravenous pentobarbital 3 days after resuscitation.

### 2.3 Animal groupings

There were 76 rabbits in this study, among which 10 rabbits were subjected to a sham operation and 66 rabbits received CA/CPR. Thereafter, 9 rabbits failed to achieve ROSC, and the remaining 57 rabbits with CA/CPR were subsequently used. The resuscitated rabbits were grouped as follows: (a) in the sham group, the rabbits were subjected to a sham operation and injected with normal saline; (b) in the CA/CPR group, the rabbits underwent CA/CPR induction and received injections of normal saline; (c) in the CA/CPR + Ang-(1-7) group, the rabbits underwent CA/CPR induction and then received subcutaneous injections of Ang-(1-7) solution (576 μg/kg/day; HY-12403, MedChemExpress, Shanghai, China) immediately after resuscitation; (d) in the CA/CPR + Ang-(1-7) + A779 group, the rabbits underwent CA/CPR induction and received subcutaneous injections of Ang-(1-7) and A779 solution (a MasR antagonist; 576 μg/kg/day; HY-P0216, MedChemExpress) immediately after resuscitation; (e) in the CA/CPR + LY294002 group, the rabbits underwent CA/CPR induction and received intraperitoneal injections of 0.6 mg/kg LY294002 (an inhibitor of PI3K; HY-10108, MedChemExpress) immediately after resuscitation; and (f) in the CA/CPR + Ang-(1-7) + LY294002 group, the rabbits underwent CA/CPR induction and were administered Ang-(1-7) solution (576 μg/kg/day) and 0.6 mg/kg LY294002. The Ang-(1-7) and A779 doses were chosen as described in a previous study [33]. The dose of LY294002 was also selected as previously described [34]. The CA/CPR groups had ten rabbits each. Subcutaneous and intraperitoneal injections were administered twice per day for 3 days.

### 2.4 Neurological functional assessment

The neurological deficit score (NDS) was assessed at 24, 48, and 72 h after ROSC, as shown in Table 1 [35]. The evaluation included five aspects. Functional assessment was conducted by two investigators in a blinded manner, and the maximum score was 100, representing normal conditions.

### 2.5 Brain water content

After the rabbits were euthanized, the brains were quickly removed, wiped clean using filter paper, and weighed to obtain the wet weight. Thereafter, the tissues were dried at 70°C overnight to obtain the dry weight.

\[\text{Brain water content}( \% )\text{}=\text{}(\text{wet weight - dry weight})\hspace{11.2em}/\text{wet weight}\times 100 \% .]\]

### 2.6 ELISA

At 72 h after ROSC, blood samples were extracted from the tail vein of each rabbit in the sham, the CA/CPR, the CA/CPR + Ang-(1-7), and the CA/CPR + Ang-(1-7) + A779 groups in vacutainer tubes (BD Biosciences, Shanghai, China) and centrifuged at 1,300×*g* for 10 min at 4°C to obtain the supernatant serum. Concentration changes in serum S100 calcium-binding protein B (S100B), neuro-specific enolase (NSE), Ang II, and Ang-(1-7) were evaluated using commercially available ELISA kits according to the manufacturer’s instructions. ELISA kits used in this study included S100B ELISA kit (SP26548, Saipei Biotechnology Co., Ltd, Wuhan, China), NSE ELISA kit (SP26202, Saipei Biotechnology Co., Ltd), Ang II ELISA kit (SP26282, Saipei Biotechnology Co., Ltd), and Ang-(1-7) ELISA kit (ml028050, Enzyme-linked Biotechnology Co., Ltd, Shanghai, China).

### 2.7 Western blotting

The hippocampal CA1 region was separated from brain tissues of rabbits from the sham, the CA/CPR, the CA/CPR + Ang-(1-7), the CA/CPR + Ang-(1-7) + A779, CA/CPR + LY294002, and CA/CPR + Ang-(1-7) + LY294002 groups and homogenized in precooled RIPA lysis buffer (20-188, Sigma‒Aldrich, Shanghai, China) with protease inhibitors (K1007, ApexBio, Shanghai, China,) followed by 10 min of centrifugation at 12,000×*g* at 4°C. The protein concentration was quantified by a BCA protein assay kit (20201ES86, Yeasen, Shanghai, China). Protein samples (40 μg) were resolved on sodium dodecyl sulfate-polyacrylamide gel electrophoresis gels and transferred onto polyvinylidene fluoride membranes. After being blocked with 5% nonfat milk, the membranes were incubated overnight with primary antibodies against ACE (ab216476, 1:100; Abcam), phosphorylated PI3K (ab182651, 1:1,000; Abcam), ACE2 (AF2335, 1:2,500; Beyotime, Shanghai, China), Akt (ab89402, 1:2,500; Abcam), β-actin (ab8227, 1:3,000; Abcam), AT1R (ab59015, 0.2 μg/mL; Abcam), AT2R (ab254561, 0.5 μg/mL; Abcam), MasR, PI3K (ab191606, 1:1,000; Abcam), phosphorylated Akt (ab338449, 1:1,000; Abcam), cleaved caspase-9 (#9507, 1:1,000; Cell Signaling Technology [CST], Shanghai, China), cleaved caspase-3 (#9664, 1:1,000; CST), and cytochrome c (ab90529, 1 μg/mL; Abcam) at 4°C followed by incubation with secondary antibodies for 1 h at room temperature. Proteins were visualized by enhanced chemiluminescence reagents (36222ES76, Yeasen). The immunoblot images were evaluated using ImageJ software (NIH, Bethesda, MD, USA).

### 2.8 Terminal deoxynucleotidyl transferase dUTP nick end labeling (TUNEL) staining

The brains were collected 72 h after resuscitation from each rabbit in the sham, the CA/CPR, the CA/CPR + Ang-(1-7), and CA/CPR + Ang-(1-7) + LY294002 groups and cut at the hippocampal level. To detect nuclear DNA fragmentation in apoptosis, TUNEL assay was conducted with a TUNEL Apoptosis Detection Kit (40308ES50, Yeasen). The paraffin-embedded brain tissues were cut into slices (5 mm) and then dewaxed, rehydrated, and rinsed. Then, apoptosis in hippocampal neurons was assessed using the TUNEL kit at 37°C for 30 min followed by incubation in DAPI solution (Solarbio, Beijing, China) for 5 min for nuclear staining in the dark. Finally, three fields of view in the hippocampus CA1 region were randomly selected for each section, and the number of TUNEL-positive cells were counted using a fluorescence microscope (Olympus, Tokyo, Japan) with a 40×/0.65 objective. Image analysis was performed in a double-blind manner.

### 2.9 Statistical analysis

The data were analyzed by GraphPad Prism (GraphPad Inc., San Diego, CA, USA) and are reported as the mean ± standard deviation. One-way analysis of variance followed by Tukey’s *post hoc* analysis and Student’s *t* test were used for statistical analysis. A value of *p* < 0.05 was considered statistically significant.


**Ethical approval:** The research related to animals’ use has been complied with all the relevant national regulations and institutional policies for the care and use of animals. All animal studies were conducted under the Guide for the Care and Use of Laboratory Animals of the National Institutes of Health, and the experimental protocol was approved by the Ethics Committee of Wuhan Myhalic Biotechnology Co., Ltd (approval number: 202207127).

## 3 Results

### 3.1 Effects of MasR activation or inhibition on neurological function and brain damage following CA/CPR

**Figure 1:**
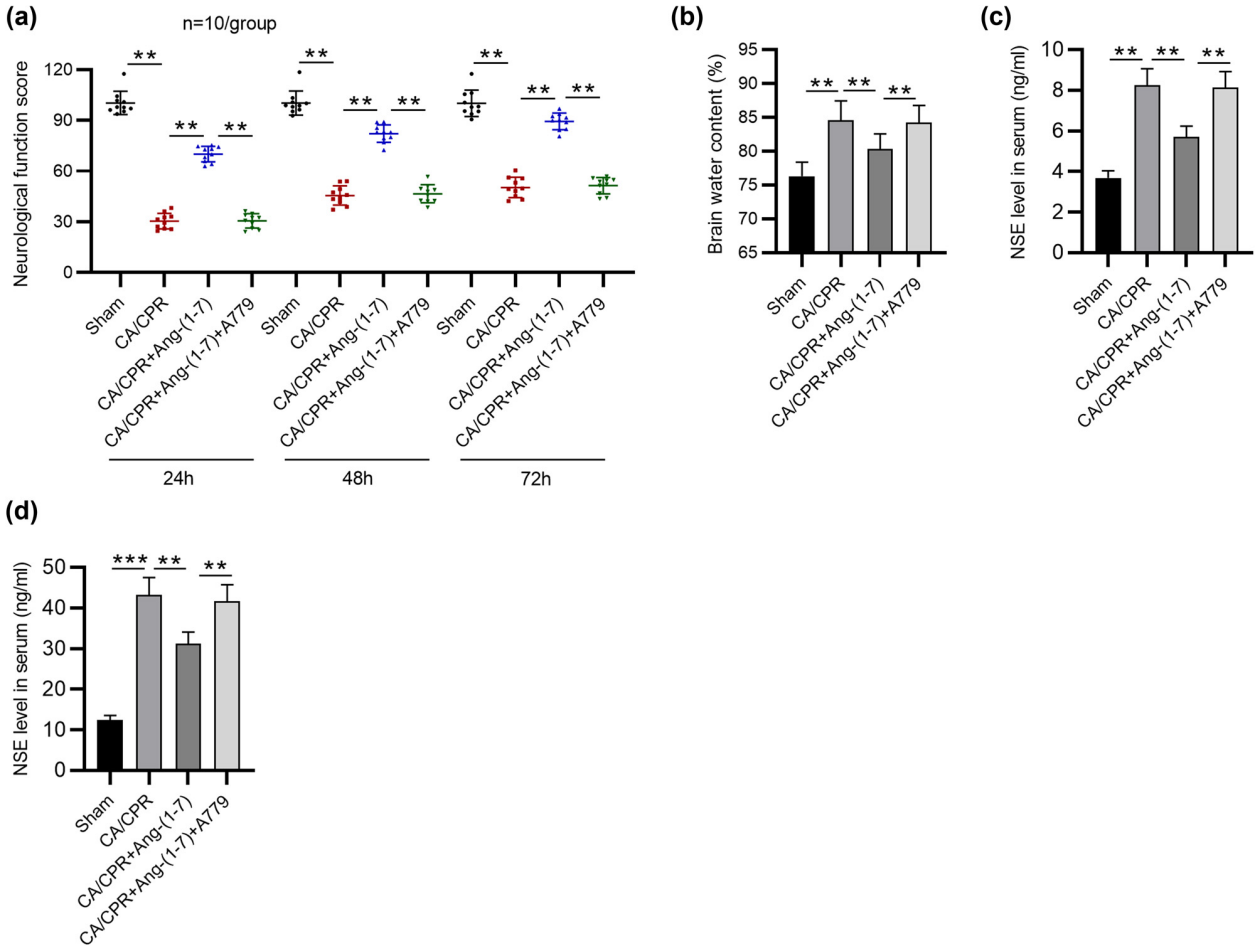
Effects of MasR activation or inhibition on neurological deficits and brain damage caused by CA/CPR. (a) Assessment of the NDS (*n* = 10 per group). (b) Detection of brain water content (*n* = 5 per group). (c) and (d) Serum levels of NSE and S100B 72 h after CA/CPR or sham operation were evaluated by ELISA (*n* = 5 per group). **p < 0.01.

The NDS was significantly decreased following CA/CPR induction, which was improved by Ang-(1-7) administration, whereas A779 administration in the presence of Ang-(1-7) significantly increased the NDS (Figure 1a). Then, brain water content was measured, which was increased after CA/CPR; Ang-(1-7) reversed this increase, whereas A779 attenuated the inhibitory effect of Ang-(1-7) (Figure 1b). NSE is an intracellular enzyme located within neurons and neuroectodermal cells, and S100B protein is located in astrocytes and Schwann cells [36]. Increases in NSE and S100B indicate neuronal damage [37]. Ang-(1-7) administration abolished the increase in serum NSE and S100B levels at 72 h after resuscitation, whereas A779 treatment increased the serum levels of NSE and S100B in the context of Ang-(1-7) administration (Figure 1c and d). Overall, MasR activation alleviates CA/CPR-induced neurological deficits, brain edema, and neuronal damage, and its inhibition has the opposite effects.

### 3.2 Effects of MasR activation or inhibition on the ACE/Ang II/AT1R axis

**Figure 2:**
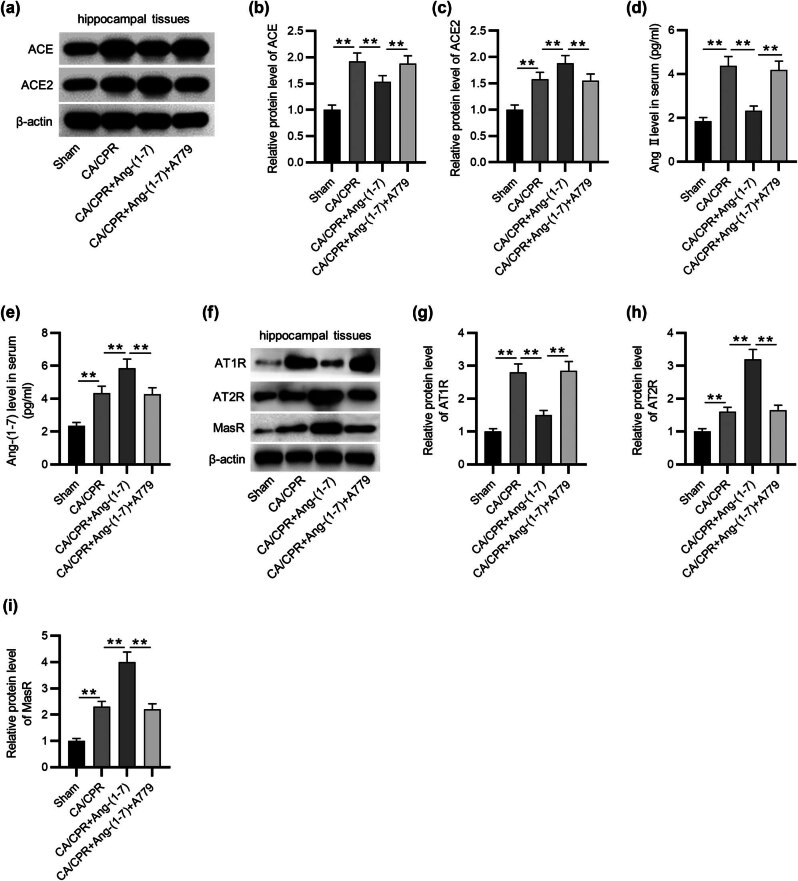
Effects of MasR activation or inhibition on the ACE/Ang II/AT1R axis. (a)–(c) Western blot analysis of ACE and ACE2 protein levels. (d) and (e) ELISA of Ang II and Ang-(1-7) serum levels. (f)–(i) Western blot analysis of AT1R, AT2R, and MasR protein levels. *n* = 5 per group in all experiments. **p < 0.01.

Western blotting demonstrated that ACE protein levels were markedly augmented post-CA/CPR, which was decreased by Ang-(1-7) administration. However, A779 treatment limited the inhibitory effect of Ang-(1-7) on the protein levels of ACE. Additionally, Ang-(1-7) remarkably increased ACE2 protein expression, whereas A779 administration reversed the enhancing effect of Ang-(1-7) on the protein level of ACE2 (Figure 2a–c). The ELISA results revealed that Ang-(1-7) administration decreased Ang II serum expression, while A779 had the opposite effect (Figure 2d). Besides, Ang-(1-7) administration effectively elevated Ang-(1-7) serum expression, whereas A779 decreased Ang-(1-7) expression after CA/CPR (Figure 2e). Moreover, the protein level of AT1R was decreased by Ang-(1-7) administration, whereas A779 rescued the decreased AT1R expression caused by Ang-(1-7) in CA/CPR-treated rabbits. Meanwhile, Ang-(1-7) injection obviously upregulated AT2R and MasR protein levels, whereas A779 administration reduced AT2R and MasR protein levels in the context of Ang-(1-7) after CA/CPR, as shown by western blotting (Figure 2f–i). Collectively, ACE2/Ang-(1-7)/MasR signaling inactivates the ACE/Ang II/AT1R axis.

### 3.3 Effects of MasR activation or inhibition on the PI3K/Akt pathway

**Figure 3:**
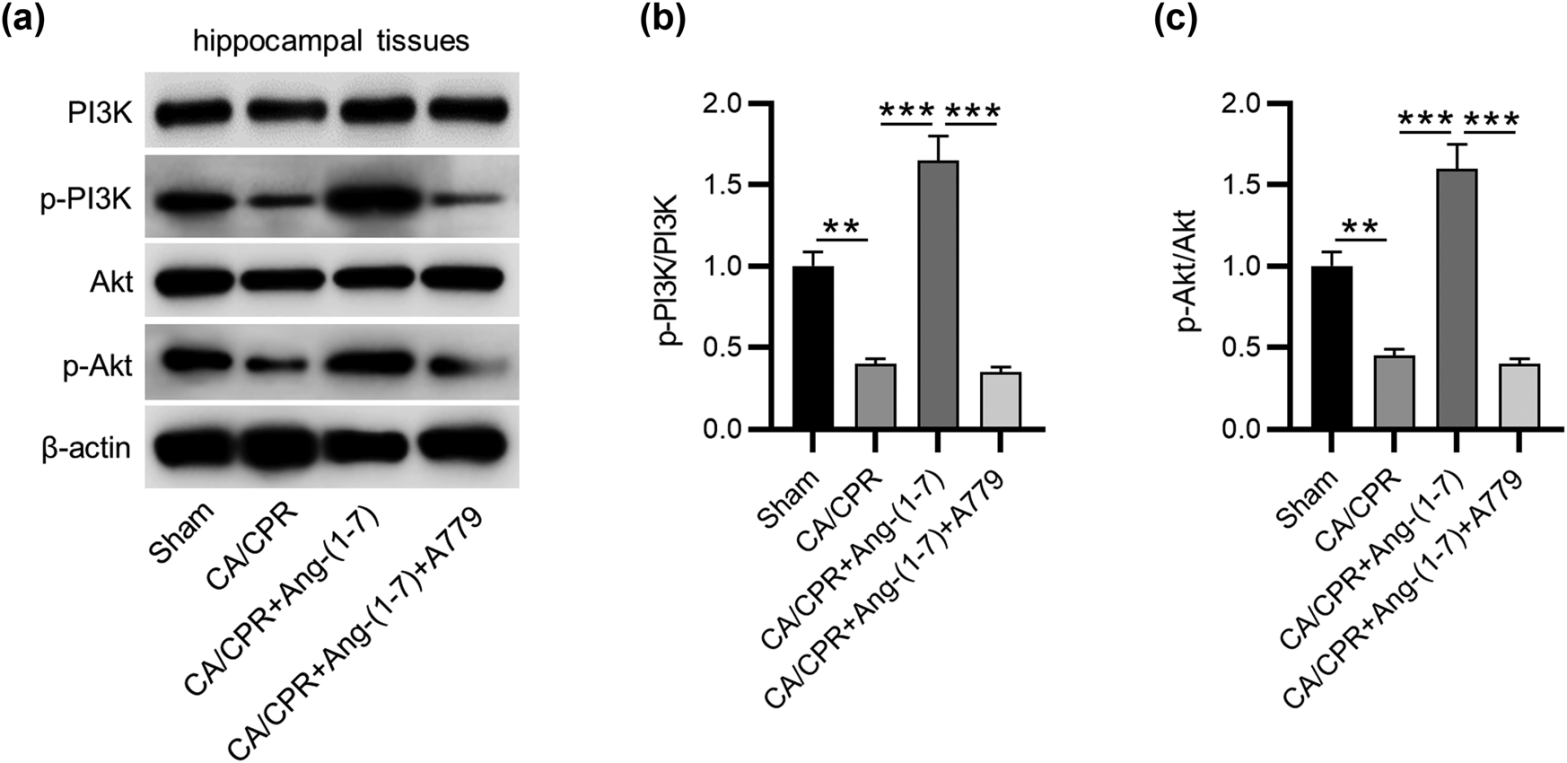
Effects of MasR activation or inhibition on PI3K/Akt signaling. (a) Blots showing PI3K, phosphorylated PI3K, Akt, and phosphorylated Akt (*n* = 5 per group). (b) and (c) Quantification of PI3K and Akt phosphorylation. **p < 0.01, ***p < 0.001.

CA/CPR-mediated inhibition of PI3K and Akt phosphorylation was abolished by Ang-(1-7) administration in CA/CPR-induced rabbits, while A779 treatment prevented this phosphorylation, suggesting that an increase in MasR could activate PI3K/Akt signaling, while MasR inhibition could inactivate this pathway (Figure 3a–c).

### 3.4 Inhibition of PI3K/Akt pathway reverses the protective effects of MasR activation against brain damage after CA/CPR

**Figure 4:**
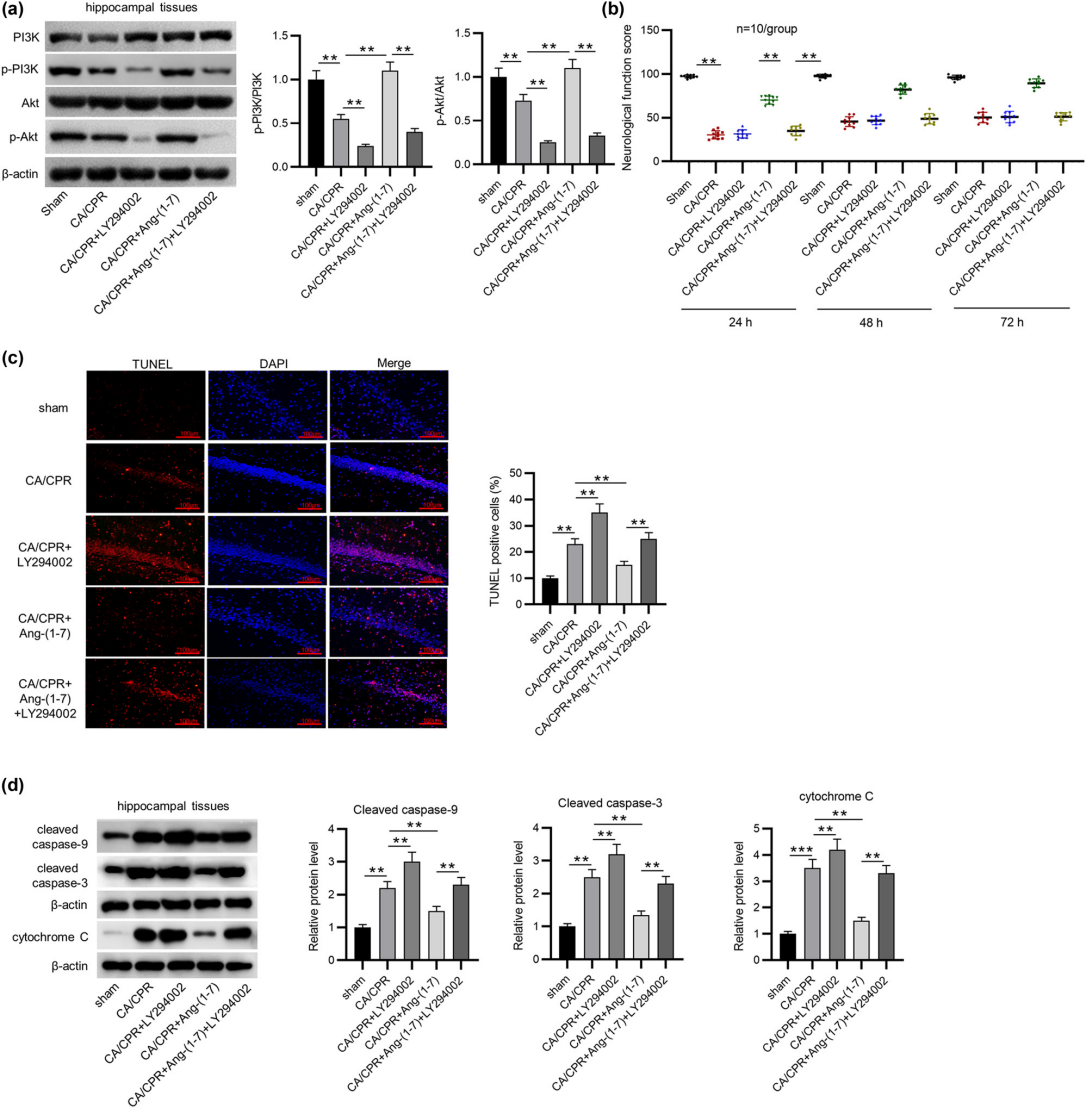
Inhibiting PI3K/Akt signaling reversed the Ang-(1-7)-induced attenuation of brain damage after CA/CPR. (a) Western blot analysis of PI3K, phosphorylated PI3K, Akt, and phosphorylated Akt protein levels (*n* = 5 per group). (b) NDS 24 h after the operation (*n* = 10 per group). (c) TUNEL staining showing neuronal apoptosis in the hippocampus (*n* = 5 per group). (d) Western blot analysis of cleaved caspase-9, cleaved caspase-3, and cytochrome C protein levels (*n* = 5 per group). **p < 0.01.

Western blotting showed that LY294002 effectively inhibited PI3K and Akt phosphorylation in the context of MasR activation in CA/CPR-induced rabbits (Figure 4a). Then, the NDS was estimated, and the data revealed that the Ang-(1-7)-induced significant increases in the NDS in CA/CPR-treated rabbits were restored by LY294002 administration (Figure 4b). Next, TUNEL assays showed that CA/CPR induction enhanced hippocampal neuronal apoptosis, which was further exacerbated after LY294002 administration, while Ang-(1-7) treatment alleviated neuronal apoptosis. However, LY294002 prevented Ang-(1-7)-mediated protection against neuronal apoptosis (Figure 4c). The release of cytochrome C can stimulate the assembly of apoptotic body containing caspase-9, thereby activating caspase-3 and causing cell apoptosis [38]. Western blotting revealed that cleaved caspase-9, cleaved caspase-3, and cytochrome C protein levels were elevated following CA/CPR, and LY294002 administration further increased these protein levels. However, Ang-(1-7) mitigated the promotion of the effects of CA/CPR, which were counteracted by LY294002 (Figure 4d). Collectively, the ACE2/Ang-(1-7)/MasR axis alleviated brain damage by attenuating hippocampal neuronal death by activating PI3K/Akt signaling.

## 4 Discussion

CA accounts for 15% of all mortality, and only 20% of discharged patients survive [39]. Accumulating evidence has suggested the critical role of hippocampal neuronal apoptosis in CA/CPR-induced brain injury [40,41]. In the current study, we used well-validated rabbit CA/CPR models [42–44], and investigated the roles of ACE2/Ang-(1-7)/MasR in CA/CPR-induced hippocampal neuronal apoptosis and its related mechanisms. The ACE2/Ang-(1-7)/MasR axis can alleviate brain injury after CA/CPR in rabbits via activation of PI3K/Akt signaling as indicated by the following findings: (a) Ang-(1-7) alleviates the CA/CPR-induced neurological deficits, brain edema, and neuronal damage, whereas A779 had the opposite effects; (b) Ang-(1-7) inactivated the ACE/Ang II/AT1R axis in CA/CPR-induced rabbits, whereas A779 reversed the suppressive effect of Ang-(1-7) on the activation of ACE/Ang II/AT1R; (c) Ang-(1-7) activated PI3K/Akt signaling, whereas A779 inactivated this pathway; and (d) inhibition of PI3K/Akt signaling reversed the protective effects of Ang-(1-7) against brain damage after CA/CPR.

Apoptosis is involved in neurological dysfunction during brain injury [45,46]. Chang et al. suggested that short-term hypoxia could attenuate myocardial apoptosis by upregulating MasR expression [47]. Xu et al. showed that MasR activation could ameliorate ventricular dysfunction and increase cardiac contractility by blocking apoptosis and inflammation [48]. Xiao et al. showed that an increase in MasR prevented the Ang-II-induced cerebral endothelial cell apoptosis [49]. Here, we showed that Ang-(1-7) administration upregulated ACE2 and MasR levels in the hippocampus and alleviated neuronal apoptosis in experimental models of CA/CPR, as evidenced by a decrease in TUNEL-positive neurons and reductions in caspase-9, caspase-3, and cytochrome C levels. Additionally, MasR activation improved the NDS, decreased brain water content, and downregulated NSE and S100B serum levels after CA/CPR, indicating that an increase in MasR could alleviate neurological deficits and neuronal damage following CA/CPR. Stegbauer et al. indicated that MasR inhibition exacerbated atherosclerosis and abdominal aortic aneurysm rupture via its apoptosis, inflammation, and oxidative stress enhancing actions [50]. Meng et al. revealed that MasR blockade exacerbated cardiac injury, thereby worsening apoptotic, fibrogenic, and inflammatory responses [51]. The current study showed that A779 (MasR antagonist) administration exacerbated brain damage and neurological dysfunction after CA/CPR.

Inactivation of PI3K/Akt signaling can increase neuronal apoptosis and intensify brain injury [52,53]. Stimulation of the ACE2/Ang-(1-7)/MasR axis can relieve Alzheimer’s disease symptoms by activating the PI3K/Akt pathway [54]. The antiparkinsonian function of Ang-(1-7) is associated with the activated PI3K/Akt pathway [55]. Here, we found that Ang-(1-7) increased PI3K and Akt phosphorylation, while A779 treatment had the opposite effects, indicating that stimulating the ACE2/Ang-(1-7)/MasR axis could activate the PI3K/Akt pathway, which is similar to the findings of previous studies.

Collectively, the ACE2/Ang-(1-7)/MasR axis can attenuate brain injury after CA/CPR by reducing hippocampal neuron apoptosis by activating PI3K/Akt signaling. This study had some limitations. First, we only used rabbits to evaluate the effect of the axis, and human subjects and other species are required for further experiments. Second, more underlying mechanisms by which this axis alleviates brain injury need to be investigated. Despite these limitations, we believe that the ACE2/Ang-(1-7)/MasR axis is a potential target for treating brain injury after CA/CPR.
