# Corrigendum to “The ACE2/Ang-(1-7)/MasR axis alleviates brain injury after cardiopulmonary resuscitation in rabbits by activating PI3K/Akt signaling”

**DOI:** 10.1515/tnsci-2022-0986

**Published:** 2024-08-06

**Authors:** Jing Cheng, Hong Yang, Fang Chen, Li Qiu, Fang Chen, Yanhua Du, Xiangping Meng

**Affiliations:** Department of Emergency, Wuhan Fourth Hospital, Wuhan 430030, China; General Practice Ward, Wuhan Fourth Hospital, No. 473 Hanzheng Street, Qiaokou District, Wuhan 430030, Hubei, China

Corrigendum to J. Cheng, H. Yang, F. Chen, L. Qiu, F. Chen, Y. Du and X. Meng. The ACE2/Ang-(1-7)/MasR axis alleviates brain injury after cardiopulmonary resuscitation in rabbits by activating PI3K/Akt signaling. Transl Neurosci. 2024;15(1):20220334. https://doi.org/10.1515/tnsci-2022-0334.

After publishing this article, the authors found the errors in the article text, which are corrected as follows:

1. The scoring criteria in Table 1 were wrongly written. The correct version is given below.

**Table 1:** Rabbit neurological deficit grading scale

		Score
1. General behavioral deficit (worst, 40 points)		
Consciousness	Attempt to explore spontaneously	20
	No attempt to explore spontaneously	0
Respirations	Normal	20
	Abnormal	0
2. Cranial nerve reflexes deficit (worst, 20 points)		
Olfactory (sniffing food)	Present	4
	Absent	0
Vision (follows hand)	Present	4
	Absent	0
Corneal	Present	4
	Absent	0
Whisker movement	Present	4
	Absent	0
Hearing (turns to clapped hands)	Present	4
	Absent	0
3. Motor deficit (worst, 10 points)		
Legs/tail movement	Normal	10
	Stiff	5
	Paralyzed	0
4. Sensory deficit (worst, 10 points)		
Legs/tail (on pinching)	Present	10
	Absent	0
5. Coordination deficit (worst, 20 points)		
Travel ledge (placed on beam)	Present	5
	Absent	0
Placing test (front paws reaching when lifted from ground by tail)	Present	5
	Absent	0
Righting reflex (attempting to right self when placed on back)	Present	5
	Absent	0
Stop at edge of table	Present	5
	Absent	0

Total score: 0 = brain dead, 100 = normal.

2. The caption of Figure 1 was incorrect, which should be changed to: The NDS was significantly decreased following CA/CPR induction, which was improved by Ang-(1-7) administration, whereas A779 administration in the presence of Ang-(1-7) significantly reduced the NDS (a). Then, brain water content was measured, which was increased after CA/CPR; Ang-(1-7) reversed this increase, whereas A779 attenuated the inhibitory effect of Ang-(1-7) (1b). NSE is an intracellular enzyme located within neurons and neuroectodermal cells, and S100B protein is located in astrocytes and Schwann cells [36]. Increases in NSE and S100B indicate neuronal damage [37]. Ang-(1-7) administration abolished the increase in serum NSE and S100B levels at 72 h after resuscitation, whereas A779 treatment increased the serum levels of NSE and S100B in the context of Ang-(1-7) administration (c and d). Overall, MasR activation alleviates CA/CPR-induced neurological deficits, brain edema, and neuronal damage, and its inhibition has the opposite effects.

3. The ordinate of Figure 1d should be changed to S100B.

The authors would like to apologize for any inconvenience caused.

